# Evaluation of flexural strength, degree of conversion, and demineralization-prevention properties in adjacent tooth structures of an experimental fissure sealant containing nano-calcium-phosphate compounds

**DOI:** 10.1186/s12903-023-03617-4

**Published:** 2023-11-22

**Authors:** Farnoosh Fallahzadeh, Maryam Pirmoradian, Solmaz Mohammadzadeh Ghasemi, Maryam Mortazavi

**Affiliations:** 1 Department of Operative Dentistry, Faculty of Dentistry, Qazvin University of Medical Sciences, Qazvin, Iran; 2 Department of Dental Biomaterials, School of Dentistry/Research Center for Science and Technology in Medicine, University of Medical Sciences, Tehran, Iran; 3 Dental Caries Prevention Research Center, Qazvin University of Medical Sciences, Qazvin, Iran; 4 Department of Restorative Dentistry, School of Dentistry, Birjand University of Medical Sciences, Birjand, Iran

**Keywords:** Hydroxyapatite, Pit and fissure sealant, Remineralization

## Abstract

**Background:**

The present study aimed to evaluate the flexural strength, degree of conversion, and demineralization-prevention ability of an experimental fissure sealant containing nano-calcium-phosphate compounds.

**Methods:**

An experimental sealant was formulated using silica and nano hydroxyapatite filler particles. The control group consisted of the DENU Seal (*n* = 10, each group). The flexural bond strength was evaluated by UTM. DC was evaluated by FTIR. To evaluate the demineralization-prevention ability, Cl V cavities in 10 third molar teeth restored with two sealant products, followed by an acid challenge then the Vickers microhardness test was carried out.

**Results:**

The mean flexural strength in the commercial group was higher than the experimental group. However, the mean flexural modulus was not significantly different between the two groups. In the experimental group, DC was significantly higher than the commercial group. Adjacent to the interface, the decrease in microhardness in the experimental group was significantly less than the commercial group. However, on the tooth surface, there were no significant differences between the two groups. In the experimental group, the decrease in microhardness at the interface was less than at the tooth surface, however the situation was opposite in the commercial group.

**Conclusions:**

Incorporating hydroxyapatite into the sealant structure might prevent demineralization, without adverse effects on flexural modulus and degree of conversion.

## Background

The pits and fissures of permanent molars are the most susceptible areas to dental caries. Preventive treatments, such as pit and fissure sealant therapy, can fill the areas susceptible to plaque accumulation on the occlusal surface to prevent dental caries in these areas [1]. In addition, if there are demineralized areas at the depth of fissures, they can prevent progression of caries due to their sealing ability [2]. One of the most challenging issues is the possibility of caries incidence in areas adjacent to these materials due to their incomplete bonding to the tooth structure [3].

Therefore, it is important to promote the mechanical properties of fissure sealants and introduce materials that can lead to remineralization or prevent demineralization to improve the longevity of these treatments [4].

The Hydroxypatite is a member of the large family of calcium phosphates and provides the ions necessary for remineralization. The remineralization capacity of nano-hydroxyapatite has been reported in studies that have incorporated these nanoparticles into glass ionomers and other resin compounds. The final product is considered a smart material that can release ions at high concentrations under acidic conditions [5]. They can also prevent further destruction and the decrease in mechanical properties under non-acidic and neutral conditions. In addition, the released ions can deposit on tooth structures as HAp crystals or reconstruct the damaged mineral structures [6].

Nano-particles of other calcium phosphates compounds, such as tricalcium phosphate (TCP), were added to fissure sealants and toothpaste previously [7]. But Since incorporating nHAp particles into resin products to promote remineralization is a new field of study, and these particles have not been incorporated into fissure sealants at such percentages, the present study aimed to investigate the flexural strength, degree of conversion, and the ability to prevent demineralization of tooth structure adjacent to a type of experimental fissure sealant containing nano-calcium-phosphate compounds after acidic challenges. The null hypotheses stated that:

Incorporating nano-calcium-phosphate compounds will not affect the flexural strength, elastic modulus, DC and deposition of HAp crystals.

## Materials and methods

### Synthesis of an experimental fissure sealant

The present in vitro study evaluated two fissure sealant groups: an experimental and a commercial fissure sealant (*n* = 10). According to the previous study [Development of antibacterial composite resin containing chitosan/fluoride microparticles as pit and fissure sealant to prevent caries] and considering the standard deviation of flexural strength in two groups equal to 15.24 and 13.57 respectively, also type I error (α) equal to 0.05 (Z = 1.96), the power of the study equal to 0.80 (Z = 0.84), the mean difference = 18, with the following formula, the number of 10 samples was estimated in each groups.

$$n=\frac{{\left({s}_1^2+{s}_2^2\right)}^2{\left({z}_{1-\frac{\alpha }{2}}+{z}_{1-\beta}\right)}^2}{{\left({\overline{x}}_1-{\overline{x}}_2\right)}^2}$$

Also for micro hardness, considering the same α and statistical power, the standard deviation in two groups equal to 1.29 and 1.26 respectively, the mean difference = 1.6, the number of 5 samples were required in each group.

The commercial fissure sealant (DENU Seal, Korea) was selected as the control group because it contains no remineralizing agent and according to manufacturer’s claim, fillers of this material are nanosize. Table 1 lists the compounds used to synthesize the experimental fissure sealant. Table 2 shows the comparsion of the composition of experimental and commercial fissure sealant.

**Table 1:** Comparison components used to fabricate the fissure sealants, the abbreviations, chemical formulae, and the manufacturers

The compounds used	Abbreviation	Chemical formula	Manufacturer
Bisphenol A glycerolate dimethacrylate (resin component)	Bis-GMA	C_29_H_36_O_8_	Sigma–Aldrich, Steinheim, Germany
Triethylene glycol dimethacrylate (resin component)	TEGDMA	C_14_H_22_O_6_	Sigma–Aldrich, Steinheim, Germany
Camphorquinone (resin component)	CQ	C_10_H_14_O_2_	Sigma–Aldrich, Steinheim, Germany
2-(Dimethylamino)ethyl methacrylate (resin component)	–	C_8_H_15_NO_2_	Sigma–Aldrich, Steinheim, Germany
Silica glass (filler)	–	Sio_2_	Sigma–Aldrich, Steinheim, Germany
Nano Hydroxyapatite (filler)	nHAp	Ca_5_(PO_4_)_3_(OH)	Morvabon, Tehran, Iran

**Table 2:** “Comparison” of chemical composition of commercial and experimental fissure sealants

Material	Chemical composition
DENU Seal	-Bisphenol A, glycidyl dimethacrylate, fumed silica, photo initiator, colorant [38]
Experimental fissure sealant	Bisphenol A-glycidyl methacrylate, Triethylene glycol dimethacrylate, 2-(Dimethylamino)ethyl methacrylate, Camphorquinone as photo initiator Silica glass and nano Hydroxyapatite as filler particles

In the pilot samples, to achieve viscosity and rheological properties, similar to the commercial brand, 30 wt% of the filler and 70 wt% of the resin (consisting of Bis-GMA, TEGDMA camphorquinone and tertiary amine) were calculated. Based on previous studies, 1 wt% of the whole resin component was dedicated to the initiator-accelerator system and 99 wt% to Bis GMA-TEGDMA (49.5 wt% Bis GMA, 49.5 wt% TEGDMA). The camphorquinone-tertiary amine ratio was adjusted at 3:1 [4]. So, to prepare 10 g of experimental fissure sealant, 3.456 g of Bis GMA, 3.456 g of TEGDMA, 0.046 g of tertiary amine and 0.023 g of camphorquinone were mixed.

The filler component consisted of 20% nHAp and 10% silica nanoparticles. The silica nanoparticles were silanized using γ-methacryloxypropyltrimethoxysilane [8]. Hydroxyapatite nanoparticles were prepared from calcium nitrate tetrahydrate, ammonium dihydrogen phosphate, and sodium hydroxide compounds using the precipitation method [9].

The synthesis of the experimental fissure sealant was modified from the study of Safwat EM [1]. All the synthesis procedures were carried out in a dark room under a red light. First, Bis-GMA and TEGDMA were mixed for 1 hour at a low speed (mean rpm = 18,000) to achieve a homogeneous mixture. Then, camphorquinone and tertiary amine were added, and mixing continued for another 30 minutes to finally achieve a homogeneous mixture. The filler components were added to the resin in 10 stages, followed by centrifugation (Hermle Z366 Series, Universal Centrifuge) in three 10-minute rounds to eliminate the entrapped air bubbles at 2000 rpm.

**Fig. 1:**
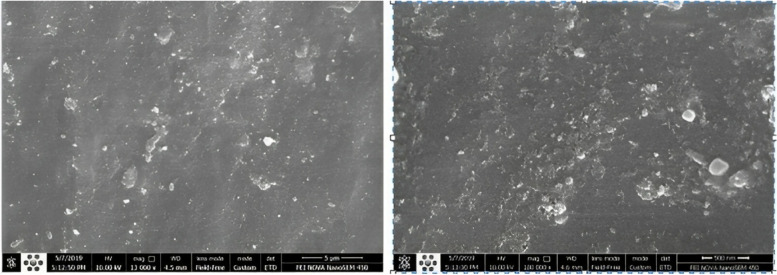
FESEM images of the synthesized fissure sealant

**Fig. 2:**
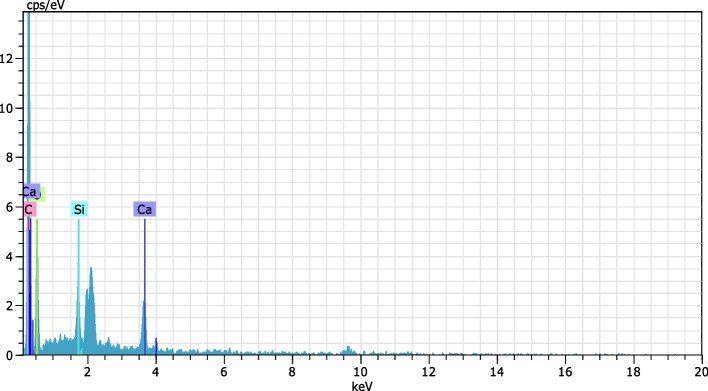
EDAX analysis of the synthesized fissure sealant

The filler particles and synthesized fissure sealant were evaluated after surface gold-sputtering using FESEM (FEI NOVA NANOSEM 450) (Figs. 1 and 2).

**Fig. 3:**
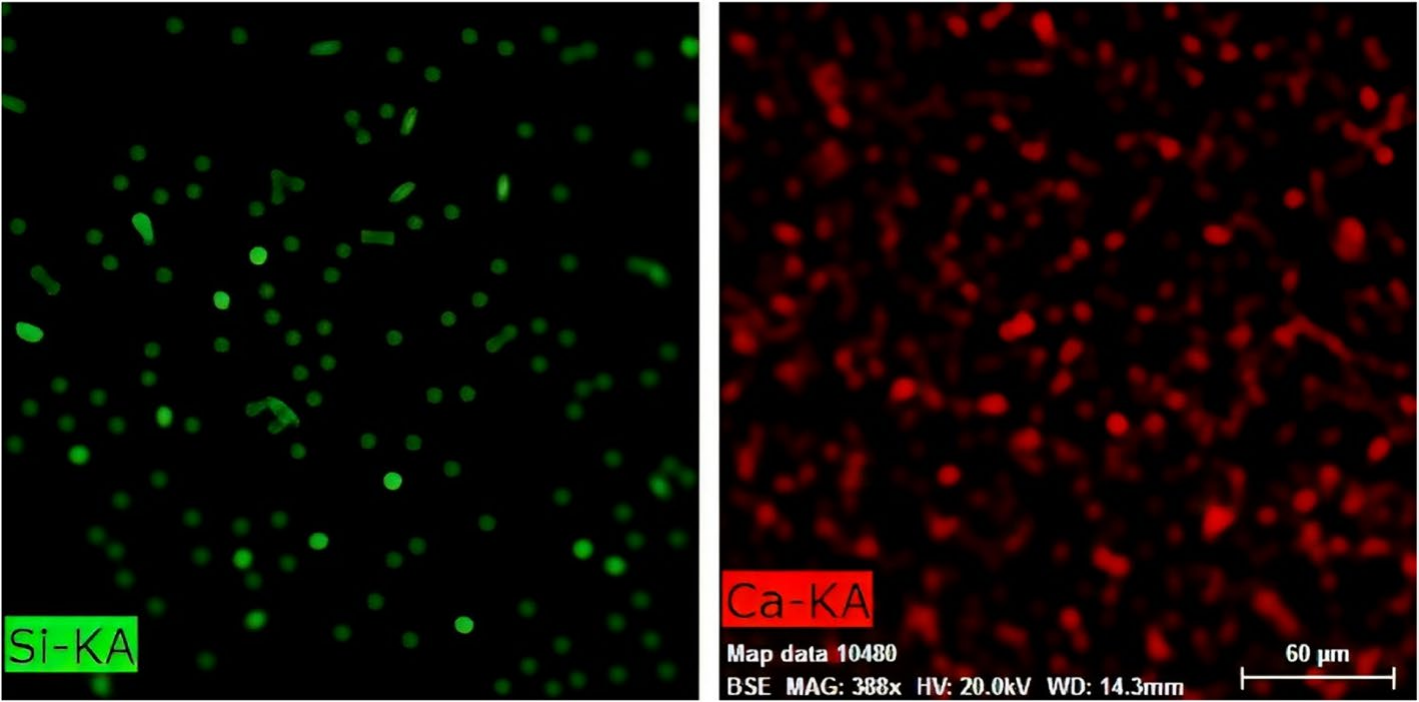
Mapping images of the synthesized fissure sealant

**Fig. 4:**
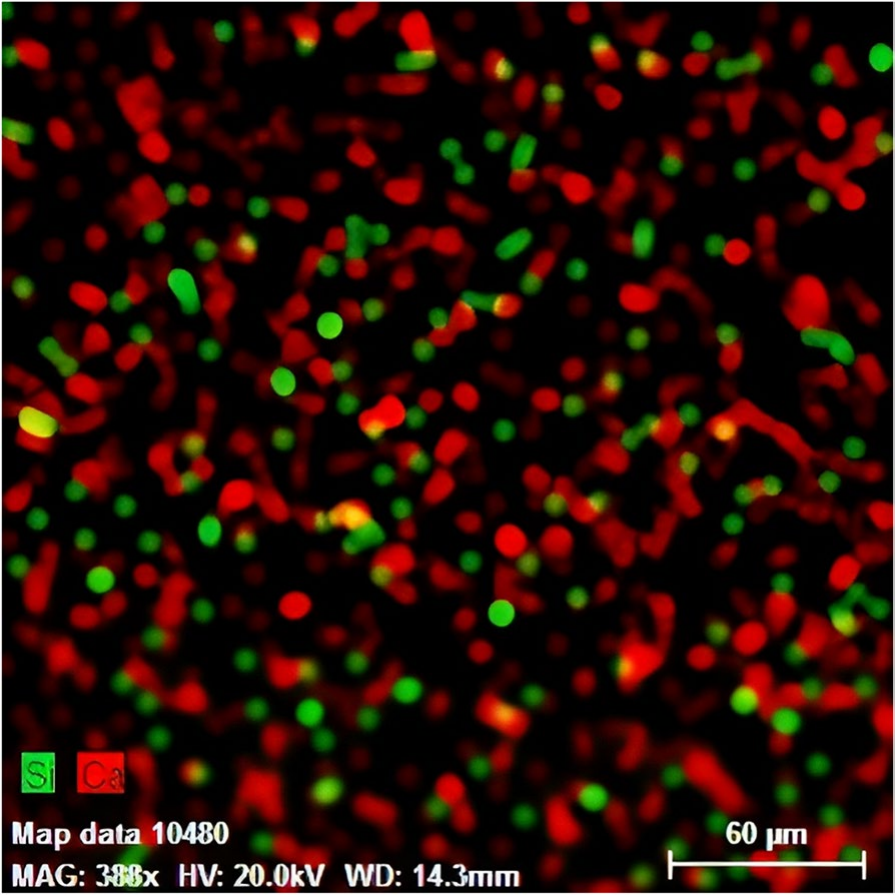
Mapping images of the synthesized fissure sealant

The images produced using the mapping technique and color-coding of Si elements (representing silica fillers) and Ca elements (representing nHAp fillers) indicated the homogeneous distribution of both components in the synthesized structure (Figs. 3 and 4). The synthesized fissure sealants were kept in a refrigerator at 4 °C until they were tested [10].

### Flexural strength and elastic modulus

Ten samples were fabricated for each group according to ISO 4049 using a stainless steel mold measuring 2 × 2 × 25 mm. The mold was placed on a glass slab; after placing the fissure sealants within the mold, the upper surface was covered with a celluloid matrix band. The sample surfaces were light-cured by overlapping in four 40-second steps using an LED light-curing unit at a light intensity of 1200 mW/cm^2^ (Blue Phase G2, Ivoclar-Vivadent). Each side were cured for 80 seconds by overlapping technique. It means that each side divided into 4 parts while each part were cured for 20 s (80 s overall). It is worth mentioning that the three sides of the specimen which were covered with metal mold were cured after removing the specimen from the mold in the same way [11].

Excess fissure sealant was removed with 200-, 1000-, and 2000-grit silicon carbide paper under running water. To ensure the completion of the curing process, the samples were stored in distilled water at 37 °C for 24 hours. Finally, the flexural strength and elastic modulus were investigated using an Instron universal testing machine (SMT-5, SANTAM, Tehran, Iran). A load cell of 20 KgF was applied at a crosshead speed of 0.5 mm/minute to the middle of the samples [8].

$$\sigma =3\textrm{FL}/2{\textrm{bh}}^2$$

where *σ*= flexural strength (MPa), F= load at the fracture point (N), L= length of the support span (mm), b= sample width (mm), and h= sample thickness (mm).

$$\textrm{E}={\textrm{FL}}^3/4{\textrm{bh}}^3\textrm{d}$$

where E = the elastic modulus (MPa), F = load at the fracture point (N), L = length of the support span (mm), b = sample width (mm), h = sample height(mm), and d = deflection at load point(mm) [8].

### Degree of conversion

A mold was made of black Plexiglass sheet with eight cavities measuring 6 mm in diameter to fabricate samples (*n* = 10, each group). The mold measured 1 mm in thickness. The mold was placed on a glass slab. After placing the fissure sealants within the mold, a celluloid matrix band was placed on the sample surfaces. Each cavity was light-cured with Blue Phase G2 (Ivoclar-Vivadent, Liechtenstein) light-curing unit at a light intensity of 1200 mW/cm^2^ for 40 s. To ensure the completion of the curing process, the samples were stored in distilled water in the incubator (DORSA, Iran) at 37 °C for 24 hours [4].

The samples were evaluated with an FTIR unit (FTIR Spectrometer, Nicolet iS10, Thermo Scientific, USA). The recorded graphs of the absorption spectra were in the 400–4000 range. Three uncured samples from each fissure sealant were evaluated in the same manner. The graphs were magnified at the 1590–1400 range using the software capacity of the machine. The absorbed peaks of aromatic C=C and aliphatic C=C bonds were identified at 1608 and 1638 wavelengths, respectively, followed by determining the areas with the Omnic software. The DC was evaluated using the following formula: [4].

$$\text{DC}\;\left(\%\right)=\left(1-\frac{\;\text{Cured}\;\left(\text{area}\;\text{under}\;1638/\text{area}\;\text{under}\;1608\right)}{\text{Uncure}\;\left(\text{area}\;\text{under}\;1638/\text{area}\;\text{under}\;1608\right)}\right)\times100$$

### Evaluation of the capacity to prevent demineralization of the adjacent tooth structures

Ten extracted human sound third molar teeth with a wide buccal surface (*n* = 5, each group) were selected. The teeth were evaluated under a stereomicroscope (Leica, Germany) at × 20 magnification. Teeth with cracks, caries or white spots, previous restorations, or hypoplastic lesions were excluded. The teeth were stored in 0.5% chloramine T solution at 4 °C in a refrigerator for 1 week then cleaned and stored in distilled water at 4 °C in a refrigerator.

Cl V cavity was prepared on the buccal surface of the teeth with a cylindrical diamond bur (008- DIA-TESSIN). Each bur was used to prepare five cavities. The cavities measured at least 4 mm in length and width and were 2 mm depth. The cavities were completely surrounded by enamel and were at least 1.5 mm away from the occlusal surface and the CEJ. The teeth were not etched because it was not possible to separate the residual effects of etching from those of pH cycling. Then the fissure sealants were placed in the cavities [12]. A celluloid matrix band was placed on the cavity surface and light-cured for 40 seconds. Then a flame-shaped 012 finishing bur (DIA-TESSIN) was used for finishing. The samples were stored in distilled water at 37 °C for 24 hours to ensure complete polymerization. Each sample served as its own control. At this point, the cavities were divided into two equal mesial and distal halves, and the mesial surface of the samples was covered with varnish. The occlusal to surface of teeth were also covered with nail varnish (Avon, Regal Red, USA) to prevent penetration of acidic compounds; the root apex was sealed with wax (Cavex, Netherland) as well. Then, pH cycling was carried out using the two solutions below:

- Preparation of 500 mL of the demineralizing solution (pH = 4–4.5): 2.0 mmol/L calcium (Ca(NO_3_)_2_·4H_2_O), 2.0 mmol/L phosphate (NaH_2_PO_4_·2H_2_O), 0.075 mmol/L acetate buffer and 0.02 ppm F.
- Preparation of 500 mL of the remineralizing solution (pH = 6.5–7): 1.5 mmol/L calcium (Ca(NO_3_)_2_·4H_2_O), 0.9 mmol/L phosphate (NaH_2_PO_4_·2H_2_O), 150 mmol/L potassium chloride, 0.03 ppm fluoride standard and 0.1 mol/L tris buffer [13].

The samples were stored for 6 hours in artificial saliva (pH = 4) as the demineralizing solution, followed by 18 hours of immersion in artificial saliva with a neutral pH as the remineralizing solution, interspersed by placing under running water when each sample was transferred from the demineralizing solution into the remineralizing solution and vice versa, for 1 minute. New solutions were used in each round [13].

After 7 days, the teeth were mounted using transparent acrylic resin and sectioned using a CNC machine at high speed horizontally and parallel to the CEJ from the middle of the cavities filled with fissure sealants. The coronal part was polished with 200-, 1000-, and 2000-grit silicon carbide paper under running water to achieve a smooth, finished surface. A Vickers hardness tester (Bareiss prufungerabau GmbH D-89610 oberdischingen, Germany) was used to apply 200 g weight for 15 seconds in four areas of each sample [14].

**Fig. 5:**
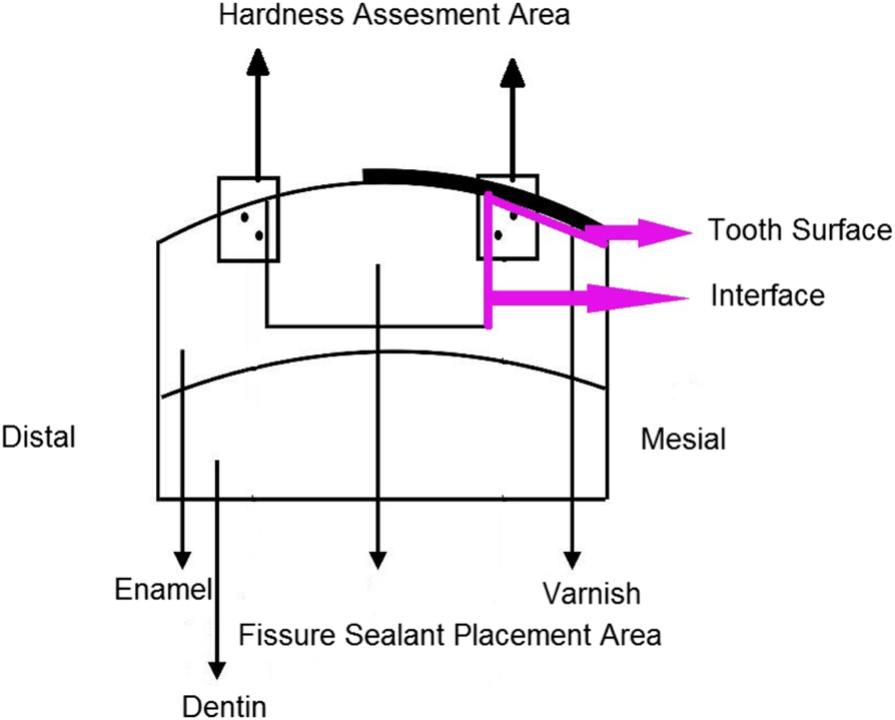
The schematic representation of the points evaluated in the microhardness test

**Fig. 6:**
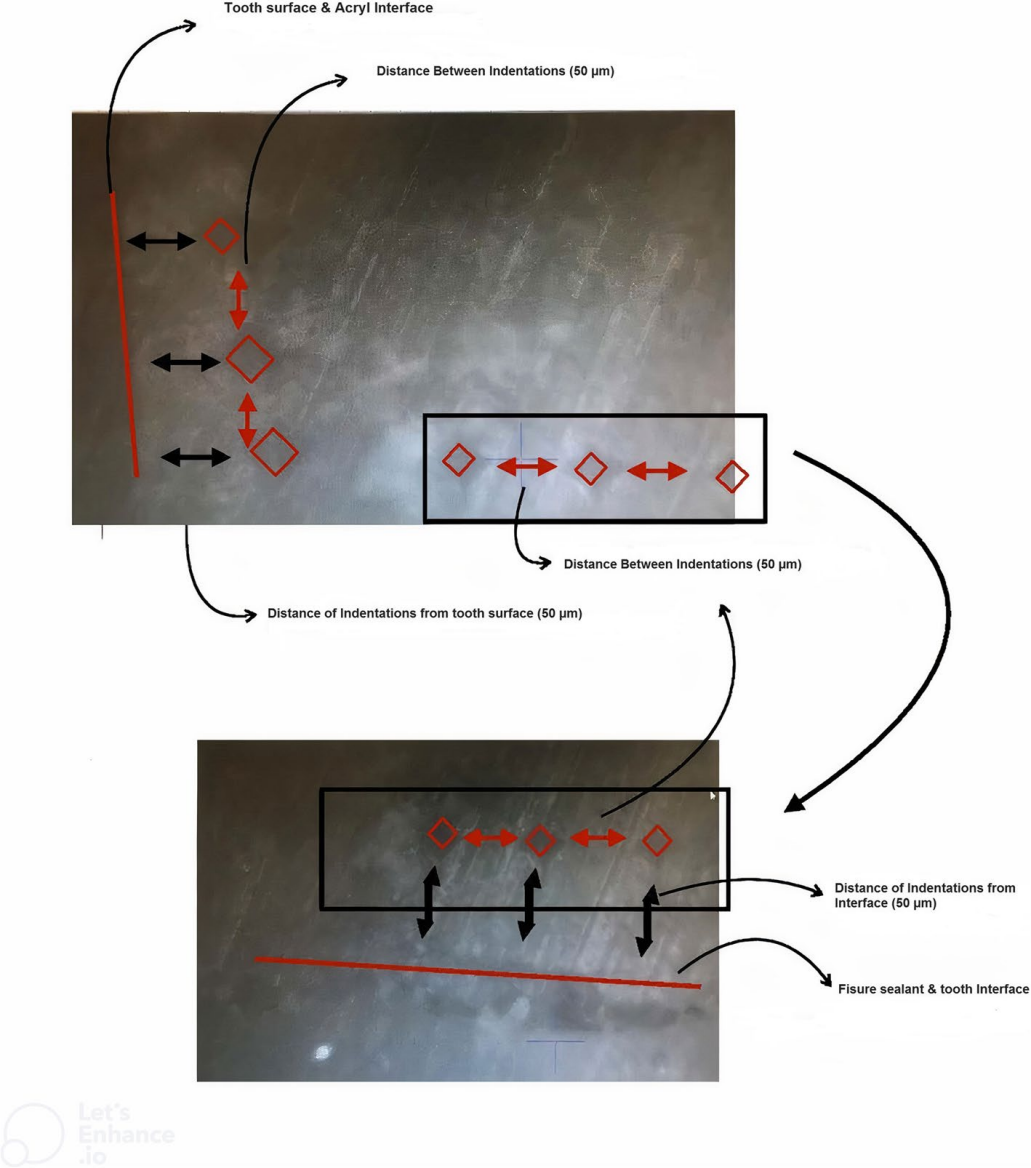
The Vickers indentations

The tooth surface and tooth–restoration interfacial area were evaluated on both the test and control sides (Fig. 5). Three indentations were made 50 μm away from the outer surface of the tooth, and three indentations were made at the tooth side, 50 μm away from the tooth/restoration interface so 6 L-shaped indentations were created in total (Fig. 6). The mean of the three values was reported as the hardness of each area [14].

SPSS 21 (IBM Corporation, NY, USA, 2012) was used for statistical analyses with independent-samples t-test (α = 0.05).

The normality of the data was checked and confirmed with the Kolmogorov-Smirnov test (*P* > 0.05).

## Results

In the present study, a commercial fissure sealant and an experimental fissure sealant were evaluated concerning their mechanical properties and their ability to prevent demineralization of the adjacent tooth structures.

### Flexural strength

**Fig. 7:**
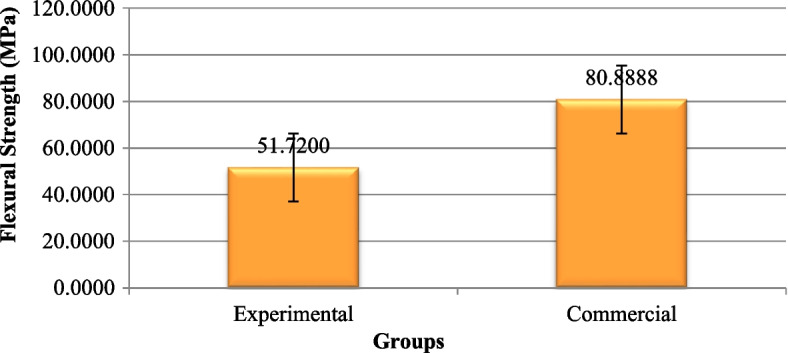
The mean flexural strength values of the experimental and commercial fissure sealants (MPa)

The mean flexural strength in the commercial group was significantly higher than the experimental fissure sealant group (*P* = 0.000) (Fig. 7)(Table 3).

**Table 3:** Comparison of mean and standard deviation values of flexural strength of the experimental and commercial fissure sealants (MPa)

Groups	Mean (MPa)	Std. Deviation	Std. Error	95% Confidence Interval for mean		*P*-value
				Lower Bound	Upper Bound	
Experimental	51.7200	6.54678	2.07028	47.0367	67.77	0.000^*^
Commercial	80.8888	8.91236	2.81833	74.5132	87.2643	

^*^statistically significant (*p* < 0.05)

### Flexural modulus

**Fig. 8:**
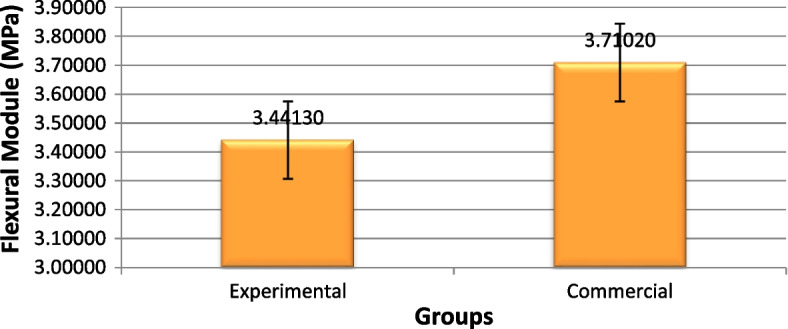
The mean elastic modulus values of the experimental and commercial fissure sealants (MPa)

According to Fig. 8, there was no significant difference in the flexural modulus between the two fissure sealant groups (*P* = 0.060) (Table 4).

**Table 4:** Comparison of mean and standard deviation values of flexural module of the experimental and commercial fissure sealants (MPa)

Groups	Mean (MPa)	Std. Deviation	Std. Error	95% Confidence Interval for Mean		*P*-value
				Lower Bound	Upper Bound	
Experimental	3.44130	0.240896	0.076178	3.26897	3.61363	0.060^*^
Commercial	3.71020	0.348137	0.110091	3.46116	3.95924	

^*^statistically significant (*p* < 0.05)

### Degree of conversion

**Fig. 9:**
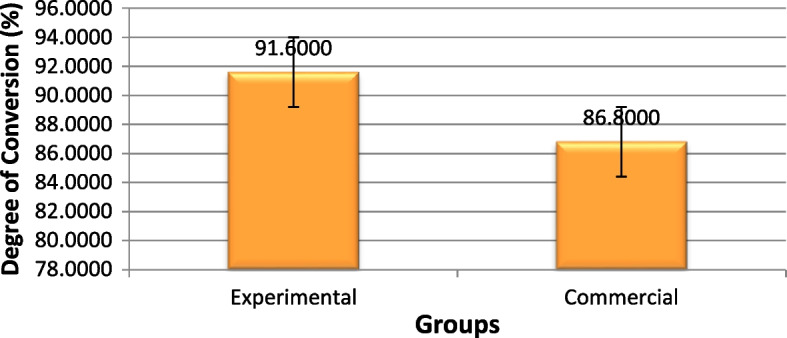
The mean values of the degree of conversion in the experimental and commercial fissure sealants (percentage)

According to the FTIR results (Fig. 9), the DC on the sample surfaces was significantly higher in the experimental fissure sealant group than the commercial fissure sealant group (*P* = 0.000) (Table 5).

**Table 5:** Comparison of mean and standard deviation values of degree of conversion of the experimental and commercial fissure sealants (%)

Groups	Mean (%)	Std. Deviation	Std. Error	95% Confidence Interval for Mean		*P*-value
				Lower Bound	Upper Bound	
Experimental	91.6000	1.14018	0.50990	90.1843	93.0157	0.000^*^
Commercial	86.8000	1.48324	0.66332	84.9583	88.6417	

^*^statistically significant (*p* < 0.05)

### Microhardness

**Fig. 10:**
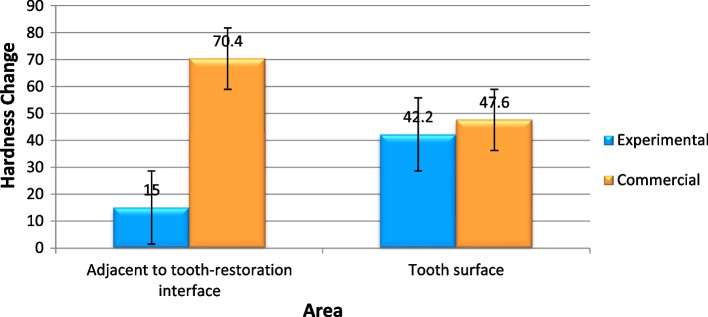
Comparison of microhardness changes (the decrease in microhardness after the acid challenge) in the experimental and commercial fissure sealants

**Fig. 11:**
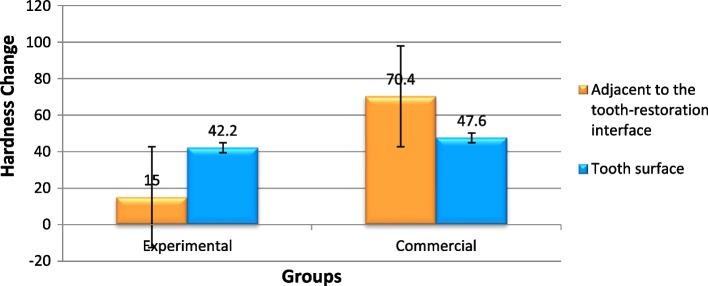
Comparison of microhardness changes (the decrease in microhardness after the acid challenge) in the area adjacent to the tooth–restoration interface and tooth surface

Figure 10 presents the results of the Vickers microhardness test. After acid exposure, adjacent to the interface, the decrease in microhardness was significantly lower in the experimental group than the commercial group. In other words, in the experimental group, after the acid exposure, microhardness did not decrease significantly; however, in the commercial group, it decreased significantly (P = 0.000). On the tooth surface, although decrease in microhardness was lower in the experimental group, the difference was not significant statistically (*P* = 0.392) (Table 6). In another comparison (Fig. 11), in the experimental group, the decrease in microhardness in the area adjacent to the interface was significantly less than the tooth surface, however, the situation was opposite in the commercial group (*P* = 0.000 in the experimental group and *P* = 0.007 in the commercial group) (Table 7).

**Table 6:** Comparison of mean and standard deviation values of hardness changes of the experimental and commercial fissure sealants

Area	Groups	Mean (befor exposure) VHN	Mean (after acid exposure) VHN	Hardness Change Mean	Std. Deviation	Std. Error	95% Confidence Interval for Mean		*P*-value
							Lower Bound	Upper Bound	
Interface area	Experimental	169.6	154.6	15.0000	4.94975	2.21359	8.8541	21.1459	0.000^*^
	Commercial	186.2	138.6	47.6000	12.30041	5.50091	32.3270	62.8730	
Surface area	Experimental	181.2	139	42.2000	5.16720	2.31084	35.7841	48.6159	0.392^*^
	Commercial	155.2	84.8	70.4000	7.30068	3.26497	61.3350	79.4650	

^*^statistically significant (*p* < 0.05)

**Table 7:** Comparison of mean and standard deviation values of hardness changes of the interface and surface area

Groups	Area	Mean (befor exposure) VHN	Mean (after acid exposure) VHN	Hardness Change Mean	Std. Deviation	Std. Error	95%Confidence Interval for Mean		*P*-value
							Lower Bound	Upper Bound	
Experimental	Interface area	169.6	154.6	15.0000	4.94975	2.21359	8.8541	21.1459	0.000^*^
	Surface	181.2	139	42.2000	5.16720	2.31084	35.7841	48.6159	
Commercial	Interface area	186.2	138.6	47.6000	12.30041	5.50091	32.3270	62.8730	0.007^*^
	Surface	155.2	84.8	70.4000	7.30068	3.26497	61.3350	79.4650	

^*^statistically significant (*p* < 0.05)

## Discussion

Nanotechnology in dentistry is attractive in two general aspects of prevention and restoration; these two aspects are pursued with the widespread use of nanoparticles [15]. nanosize agents have been incorporated into adhesive agents and restorative materials in two aspects for their antibacterial effects to prevent plaque formation and remineralization of incipient lesions [16]. nanosize materials have different chemical reactivity, bioactivity, and physical properties than their bulk forms due to their higher active surface area. Composite resins containing these particles exhibit high esthetic appearance and polishability and good wear properties [17]. Therefore, in the present study, nanoparticles were used, considering their positive characteristics.

Studies have shown that the mechanical properties of nanocomposite resins decrease with decreasing particle size, which might be attributed to the entrapment of more air bubbles within the composite resin when smaller filler particles are mixed [18, 19]. Some studies have shown improved mechanical properties after silanization of the particles. Silanization of the particles results in better integrity of nanocomposite resins, while it significantly decreases the release of ions [20]. Therefore, it has been recommended that calcium and phosphate nanoparticles be used without silanization [19]. The absence of silanization and the possible lower filler content of the experimental fissure sealant in the present study compared to the commercial fissure sealant might explain the decrease in the flexural strength in the experimental group. However, there was no significant difference in the flexural modulus between the two fissure sealants.

In the matrix component of the experimental fissure sealant in the present study, a 1:1 ratio of Bis-GMA and TEGDMA was used, similar to the chemical structure of the matrix in most resin compounds on the market. This proportion was selected to achieve proper strength, with good flowability and adequate viscosity to flow into the pits and fissures on the occlusal surfaces of the teeth [21]. Studies have reported a filler content of 35–70 vol% or 50–85% weight% of the composite resin compounds, which was decreased to 30 wt% in the present study due to low mechanical expectations from the fissure sealants [22, 23]. In the fissure sealant synthesized in the studies by Yang and Marovic, flexural strength of the synthesized fissure sealant with anticariogenic properties was lower than the control group due to the absence of silanization similar to the present study [21, 24]. According to Beun’s study, this amount of flexural strength is comparable to that of some commercial fissure sealants with ISO standards [25]. Therefore, the first null hypothesis indicating that incorporating nano-calcium-phosphate compounds will not affect the flexural strength of fissure sealants was rejected. However, the second null hypothesis that incorporating nano-calcium-phosphate compounds will not affect the elastic modulus of fissure sealants was accepted.

Evaluation with FTIR-ATR in the present study showed that in the experimental group, the degree of conversion was significantly higher than in the commercial group.

Although the filler percentage of the commercial fissure sealant was not mentioned in the catalog, considering the decrease in the flexural strength in the experimental group, it might be inferred that the experimental fissure sealant possibly had a lower filler content than the commercial product. Furthermore, it has been demonstrated that a lower filler content and the resultant lower viscosity of the resin might lead to a better distribution of the reactive groups, resulting in a higher degree of polymerization [26]. Therefore, the null hypothesis that incorporating nano-calcium-phosphate compounds will not affect the DC was rejected.

A microhardness test was used to evaluate the effects of components involved in preventing demineralization or promoting remineralization in the present study [27].. It should be noted that all the specimens were stored in 0.5% chloramine T before microhardness test and Boruziniat’ study showed chloramine T has no effect on the result of microhardness test [28]. In the present study, microhardness decreased in both groups and in both tested areas after acidic challenges, i.e., the release of ions by HAp couldn’t completely prevent demineralization after the acidic challenge; however, the experimental product containing HAp was significantly more successful in preventing demineralization in area adjacent to tooth- restoration interface.

The present study showed that demineralization in the superficial tooth structures in the experimental group was significantly higher than that at the restoration–cavity interface area; however, the situation was the opposite in the commercial group. Considering the presence of gaps measuring 10.83–64.83 nm at sealant–restoration interface reported in previous studies and no use of a bonding system in the present study, the penetration of the acidic media into the fissure sealant–tooth interface area was high [21, 29], while no irrigation and cleaning were carried out in this area in contrast to the surface area. Therefore, it is expected that the extent of demineralization at the interfacial area would be more than that in the surface area [30]. Under the clinical condition, too, in these areas, there is a high plaque accumulation and caries since surface cleaning is not possible by using mechanical systems, consistent with the findings in the commercial group [30]. However, in the experimental group, better results in the interfacial area, compared to the surface area, might confirm the positive effect of the synthesized fissure sealant and HAp fillers on the demineralization of the adjacent tooth structures. According to studies, HAp fillers added to the resin composites can exhibit antibacterial effects in aqueous media. The main mechanism of antibacterial effect of HAp is dissolution and release of calcium-phosphate ions and consequently pH increasing. In acidic condition like caries process, the release of ions increase that can help both the remineralization and antibacterial effect [31].

The hydroxyapatite used was semi crystalline, which makes it more soluble in acidic aqueous media [32]. This low crystallinity is predictable due to the synthesis mechanism of these nanofillers (without placing these compounds in oven) [33]. Under such a condition, it appears that in the surrounding acidic environment, the first compound available for dissolution is the HAp fillers of the fissure sealant. On the other hand, the nanostructure of these fillers in all the dimensions compared to the apatite whiskers in the enamel is another reason for their dissolution priority at low pH values [34]. After ion released and increasing their concentration, pH increases referred to as buffering [4]. It appears that the significant achievement with fissure sealants containing HAp nanofillers on the wall adjacent to the interface can be explained by this phenomenon because this wall was only exposed to the fluids penetrating the fissure sealant–tooth interface. In fact, the acidic media penetrating the interface are effectively neutralized by the ions released from the HAp nanofillers [4].

Another aspect is that components such as HAp and some bioglasses are highly capable of depositing mineral salts on their surface [4]. Under such a condition, it is expected that the ions deposited on these surfaces remaining by the electrostatic forces and secondary bonds, in neutral storage conditions in remineralizing solution (pH = 7), become active and drive the environments’ ion gradient at interface towards remineralization or formation of HAp crystals. Previous studies have shown that components such as bioglasses that exhibit similar behaviors in biologic media, require at least 3–6 hours (in the laboratory) to crystallize these ions [35]. Therefore, the acid challenge before this time can be neutralized by the absorbed ions (as forefront) and the fillers in this resin compound would not decrease logarithmically in each acid challenge. Materials with calcium- phosphate components, in aqueous environment can release calcium and phosphate ions which interact with the tubular fluid of dentin or with saliva around the enamel. After this reaction amorphous calcium phosphate is formed and over time crystalized and changed to Hydroxyapatite which can bond to tooth structure and serve as calcification foci [36]. This process continues by continuous absorption of large amounts of calcium and phosphate into the lesion, resulting in natural but accelerated remineralization [37]. Therefore, the fourth null hypothesis that incorporating nano-calcium-phosphate compounds will not affect the deposition of HAp crystals in the adjacent tissues under acidic challenge was rejected.

The reason for avoiding routine procedures such as acid etching in the evaluation protocols of such studies is the effect of the demineralization process due to acid etching on the amount and remineralization capacity of dental tissues [12]. Therefore, the direct and precise effect of the material cannot be evaluated, and correct data cannot be achieved regarding its function.

Considering the limitations of the present study, it is suggested that further studies be carried out using microradiograph in microhardness test and use of different types of silica containing lower rate of silanization.

## Conclusion

Within the limitations of this study, it can be concluded that adding nHAp to fissure sealants as filler particle can enhance the caries prevention capacity of these resin compounds, however, they can have an adverse effect on their mechanical properties due to lack of silanization and possibly low filler content.

## Data Availability

The datasets used and analyzed during the current study are available from the corresponding author on reasonable request.

## Abbreviations

DC: Degree of conversion
HAp: Hydroxyapatite
nHAp: Nano Hydroxyapatite
wt%: Weight Percentage
vol%: Volume Percentage
Bis-GMA: Bisphenol-A-glycidyl methacrylate
TEGDMA: Triethylene glycol dimethacrylate
FESEM: Field emission scanning electron microscopy
FTIR: Fourier transform infrared
FTIR-ATR: Fourier transform infrared spectroscopy- Attenuated total reflectance
SPSS: Statistical package for the social sciences
CEJ: Cemento- enamel junction
